# Tennessee’s Dental Law

**Published:** 1891-09

**Authors:** 


### Tennessee's Dental Law.

"A bill entitled 'An Act to Regulate the Practice of Dentistry in the State of Tennessee, and to Punish Violators thereof.'"

SECTION. 1. *Be it enacted by the General Assembly of the State of Tennessee,* That it shall be unlawful for any person to practice, or attempt to practice, dentistry or dental surgery in the State of Tennessee without first having received a diploma from some reputable dental college, school, or university department, duly authorized by the laws of this State, or some other of the United States, and in which college, school, or university department there are at the time of issuance of such diploma annually delivered a full course of lectures and instructions in dentistry and dental surgery; *Provided,* That nothing in Section 1 of this Act shall apply to any person engaged in the practice of dentistry or dental surgery in the State at the time of the passage of this Act, except as hereinafter provided. *And, provided further,* That nothing in this Act shall be so construed as to prevent physicians, surgeons or others from extracting teeth.

SEC. 2. A Board of Examiners, consisting of six practicing dentists of acknowleded ability as such, two of whom shall be residents in each of the three subdivisions of the State, East, Middle and West Tennessee, is hereby created, who shall have authority to issue certificates to persons in the practice of dentistry or dental surgery in the State at the time of the passage of this Act; and also, to decide upon the validity of such diplomas as may be subsequently presented for registration, as hereinafter provided, and issue cirtificates to all applicants who may hereafter apply to said Board and pass a satisfactory examination.

Sec. 3. The members of said Board shall be appointed by the Governor, and shall serve for a term of three years, excepting that the members of the Board first appointed shall be made as follows; Two for one year, two for two years, and two for three years, respectively, and until their successors are duly appointed. In case of vacancy occurring in said Board by resignation, removal from State, or death, such vacancy may be filled for its unexpired term by the Governor as provided by this Act.

Sec. 4. Said Board shall keep a record, in which shall be registered the names and residence, or places of business, of all persons authorized under this Act to practice dentistry or dental surgery in this State. It shall elect one of its members President and one Secretary thereof. And it shall meet at least once in each year, at the time and place fixed for the meeting of the State Dental Association, and as much oftener and at such times and places as it may deem necessary. The majority of the members of said Board shall constitute a quorum, and the proceedings thereof shall be open to public inspection.

Sec. 5. Every person engaged in the practice of dentistry or dental surgery within this State at the time of the passage of this Act shall, within six months thereafter, cause his or her name, residence and place of business to be registered with said Board of Examiners, upon which said Board shall issue to such persons a certificate duly signed by a majority of the members of said Board, and which certificate shall entitle the person to whom it is issued to all the rights and privileges set forth in Section 1 of this Act.

Sec. 6. Any person desiring to commence the practice of dentistry or dental surgery within this State after the passage of this Act, shall before commencing such practice, file for record in a book kept for such purpose, with said Board of Examiners, his or her diploma, or duly authenticated copy thereof, the validity of which said Board shall have the power to determine. If accepted, said Board shall issue to the person holding such diploma a certificate duly signed by all, or a majority of the members of said Board, and which certificate shall entitle the person to whom it is issued to all the rights and privileges set forth in Section 1 of this Act: *Provided*, That any person, whether holding a diploma aforesaid or not, shall have the privilege of makeng application to said Board, and upon undergoing a satisfactory examination, shall be entitled to a certificate in like manner as a person holding a diploma, and upon the same terms.

Sec. 7. To provide for the proper and effective enforcement of this Act, said Board of Examiners shall be entitled to the following fees, to-wit: For each certificate to persons engaged in the practice in the State at the time of the passage of this Act, the sum of one dollar; for each certificate issued to persons not engaged in the practice of dentistry in the State at the time of passage of this Act, the sum of five dollars.

Sec. 8. The members of said Examining Board shall receive the compensation of five dollars per day for each day actually engaged in the duties of his office, which, together with all other legitimate expenses incurred in the performance of such duties, shall be paid from the fees and penalties received by the Board under the provisions of this Act, and no part of the expenses of said Board shall at any time be paid out of the State Treasury. All moneys in excess of said "*per diem*" allowance and other expenses shall be held by the Secretary of said Board as a special fund for meeting the expenses of said Board, he giving such bond as the Board may from time to time direct, and said Board shall make an annual report of its proceedings to the Governor by the 15th day of December of each year, together with an account of all moneys received and disbursed by them in the pursuance of this Act.

Sec. 9. Any person who shall violate this Act by practicing or attempting to practice dentistry or dental surgery within the State, without first complying with the provisions of this Act, shall be deemed guilty of a misdemeanor, and upon conviction thereof shall be fined in a sum not less than twenty-five nor more than three hundred dollars.

Sec. 10. This Act shall take effect from and after its passage, the public welfare demanding it.

